# Incidence of delayed bleeding in patients on antiplatelet therapy after mild traumatic brain injury: a systematic review and meta-analysis

**DOI:** 10.1186/s13049-021-00936-9

**Published:** 2021-08-23

**Authors:** Giorgio Colombo, Mattia Bonzi, Elisa Fiorelli, Alessandro Jachetti, Viviana Bozzano, Giovanni Casazza, Monica Solbiati, Giorgio Costantino

**Affiliations:** 1 Pronto Soccorso e Medicina D’Urgenza, Fondazione IRCCS Ca’ Granda Ospedale Maggiore Policlinico, 20122 Milan, Italy; 2 Università degli Studi di Milano, 20122 Milan, Italy; 3 Medicina Interna Allergologia e Immunologia, Fondazione IRCCS Ca’ Granda Ospedale Maggiore Policlinico, 20122 Milan, Italy; 4 Dipartimento di Scienze Biomediche e Cliniche “L. Sacco”, Università degli Studi di Milano, Milan, Italy; 5 Dipartimento di Scienze Cliniche e di Comunità, Università degli Studi di Milano, Milan, Italy

**Keywords:** Traumatic brain injury, Intracerebral hemorrhage, Delayed intracerebral bleeding, Antithrombotic agents

## Abstract

**Background:**

The scientific evidence regarding the risk of delayed intracranial bleeding (DB) after mild traumatic brain injury (MTBI) in patients administered an antiplatelet agent (APA) is scant and incomplete. In addition, no consensus exists on the utility of a routine repeated head computed tomography (CT) scan in these patients.

**Objective:**

The aim of this study was to evaluate the risk of DB after MTBI in patients administered an APA.

**Methods:**

A systematic review and meta-analysis of prospective and retrospective observational studies enrolling adult patients with MTBI administered an APA and who had a second CT scan performed or a clinical follow-up to detect any DB after a first negative head CT scan were conducted. The primary outcome was the risk of DB in MTBI patients administered an APA. The secondary outcome was the risk of clinically relevant DB (defined as any DB leading to neurosurgical intervention or death).

**Results:**

Sixteen studies comprising 2930 patients were included in this meta-analysis. The pooled absolute risk for DB was 0.77% (95% CI 0.23–1.52%), ranging from 0 to 4%, with substantial heterogeneity (I^2^ = 61%). The pooled incidence of clinically relevant DB was 0.18%. The subgroup of patients on dual antiplatelet therapy (DAPT) had an increased DB risk, compared to the acetylsalicylic acid (ASA)-only patients (2.64% vs. 0.22%; p = 0.04).

**Conclusion:**

Our systematic review showed a very low risk of DB in MTBI patients on antiplatelet therapy. We believe that such a low rate of DB could not justify routine repeated CT scans in MTBI patients administered a single APA. We speculate that in the case of clinically stable patients, a repeated head CT scan could be useful for select high-risk patients and for patients on DAPT before discharge.

**Supplementary Information:**

The online version contains supplementary material available at 10.1186/s13049-021-00936-9.

## Background and aim of the study

Traumatic brain injury (TBI) is one of the most common reasons for visits to the Emergency Department (ED) [1, 2]. More than 90% of patients who have suffered from head trauma present with a mild traumatic brain injury (MTBI), which is usually identified by a Glasgow Coma Scale (GCS) score ≥ 13 [3–5]. MTBI is generally benign, without any sequelae; however, in almost 10% of cases, it is associated with acute intracranial hemorrhage (ICH), especially in elderly people who, for several pathophysiological reasons, are more susceptible to intracranial complications [6–9]. Although less than 1% of patients with complications require neurosurgical intervention, considering the potentially life-threatening condition of some patients, it is of pivotal importance to identify risk factors that can help clinicians select such cases and avoid useless imaging in low-risk patients. This is particularly important for reducing the observation time in the ED or the length of hospitalization, which is well known to increase the risk of complications such as infection or delirium, especially in the elderly population.

While there is agreement among several guidelines recommending a computed tomography (CT) scan after MTBI in patients administered an anticoagulant, there is no consensus on how to monitor patients on antiplatelet therapy. Apart from neurological deterioration, there is even more confusion on indications for repeating a CT scan, resulting in each institute having its own protocol; many institutes recommend a routine repeated CT scan and observation for 12–24 h in patients receiving an anticoagulant even though several studies have demonstrated a low risk of delayed bleeding (DB) in this subgroup of patients [10–12].

However, most of the studies evaluating the risk of DB in MTBI patients have not distinguished between patients on anticoagulation vs. antiplatelet therapy. Moreover, some authors have found that antithrombotic therapy is not a significant risk factor for DB when adjusted for age, with an old age being the most powerful risk factor for DB [13]. Considering the ageing population and the increasing prescription of antiplatelet and dual antiplatelet therapy (DAPT), it is important to have clear indications for this particular subgroup of patients. For this reason, we conducted a systematic review and meta-analysis to evaluate the risk of DB after MTBI in patients on antiplatelet therapy.

## Methods

### Search strategy and study selection

A systematic review and meta-analysis were conducted according to the Preferred Reporting Items for Systematic Reviews and Meta-Analyses (PRISMA) Statement [14] and the Meta-analysis of Observational Studies in Epidemiology guidelines [15]. The study protocol was designed and validated by all of the investigators before the search was started.

A systematic search on MEDLINE and EMBASE was performed from database inception to December 8, 2020. Combinations of the following terms were used: (head trauma OR brain injury OR cerebral injury OR brain trauma OR cerebral trauma OR brain contusion OR cerebral contusion OR concussion OR craniocerebral trauma) AND (antithrombotic OR platelet aggregation inhibitor OR carbasalate calcium OR aspirin OR lysine acetylsalicylate OR clopidogrel OR ticagrelor OR dipyridamole OR prasugrel OR ticlopidine OR indobufen OR thienopyridine OR antiplatelet OR acetylsalicylic acid OR salicyl*). Both prospective and retrospective studies published in English were included. Because of the high risk of bias, case reports and case series were excluded.

Inclusion criteria for studies were recruitment of patients ≥ 16 years on antiplatelet therapy (dipyridamole alone was not considered an antiplatelet therapy), with MTBI at ED presentation (defined according to the study definition), a first negative head CT scan, and the availability of a second CT scan or clinical follow-up. If data on MTBI could not be separated from moderate or severe TBI, the information was included in the study. It was preferred to increase the sensitivity and overestimate the rate of DB rather than underestimate it; therefore, a small portion of non-mild TBI cases were enrolled. Sensitivity analysis without the moderate or severe TBI studies was eventually performed, and those studies including only moderate or severe TBI were excluded.

ICH was defined as any type of intracranial bleeding (epidural, subdural, subarachnoid, or intraparenchymal hemorrhage) found at head CT scan. First CT scan was defined as the first CT scan performed in the ED, irrespective of the time lag between the trauma and CT acquisition, according to the study definition. The repeated head CT scan was defined as the second CT scan performed according to the study protocol, irrespective of the time lag between the two scans, meaning that the second CT scan could have been done routinely or as clinically indicated. The bleeding was defined as delayed if it was found after a first negative head CT scan, without any time restriction.

Two reviewers (G.C. and A.J.) independently screened all of the titles and abstracts of the retrieved articles to detect potentially eligible studies and to remove irrelevant reports. If the reviewers disagreed on a given study, it was initially included to increase the search sensitivity. Full texts of the selected articles were then obtained. Four reviewers (G.C., A.J., M.B., and E.M.F.) extracted data on the study design, inclusion and exclusion criteria, sample size, clinical characteristics of the patients, mechanism of injury, antiplatelet medication, and outcomes of interest using a predefined data extraction form. For each original study, the outcome data of only those patients corresponding to our inclusion criteria were extrapolated. Disagreements were discussed by all reviewers until a consensus was obtained. If the data could not be retrieved from the selected studies, the corresponding authors were contacted for clarification.

### Study outcomes

The primary outcome of this study was to evaluate the absolute risk of DB in MTBI patients on antiplatelet therapy. The secondary outcome was the absolute risk of clinically significant DB, defined as DB leading to death or to neurosurgical intervention after a first negative CT scan. Exploratory analyses were also performed to detect any differences in DB related to the age of the patients, to the severity of the trauma, and to the type of antiplatelet medication.

### Risk of bias assessment

Two reviewers (G.C. and A.J.) independently assessed the methodological quality of the selected articles using an adapted version of the Quality Assessment of Diagnostic Accuracy study-2 (QUADAS-2) [16]; disagreements were discussed by all reviewers until a consensus was reached. QUADAS-2 was originally created to assess the quality of diagnostic studies; an adapted version of QUADAS-2 was used as it was more suitable for our review and review question. Indeed, the PRISMA protocol is based on the patient, intervention, comparison, outcome, and study query model, which is the basis for QUADAS-2 quality assessment as well. QUADAS-2 guides the author in assessing the risk of bias and the concern regarding applicability of the study to the review question along four domains: “patient selection,” “index test,” “reference standard,” and “flow and timing.” In our modified version, the domain regarding the index test was removed, and the other three domains were slightly adapted as presented in the supplementary material. Overall, studies that received only a low risk of bias evaluation were rated as having a “low risk of bias;” studies that received at least a high risk of bias evaluation were considered as having a “high risk of bias;” if no high risk of bias was detected but “unclear” was answered to at least one question, the study was considered at “unclear risk of bias.” The same concept was applied for the applicability of studies to the review question (Additional file 1 for adapted QUADAS-2 version template).

### Data analysis

Categorical data were reported as counts and percentages. Continuous variables were presented as the mean ± standard deviation or as the median and interquartile range, based on the original study reports. For each primary study, the incidences of ICH, mortality, and neurosurgery as the proportion of events in the included patients were calculated with the 95% confidence interval (CI). Due to expected clinical heterogeneity among the included studies, all meta-analyses were performed using the DerSimonian and Laird random-effects model, after transforming the individual study proportions according to the Freeman-Tukey double arcsine transformation. The pooled estimates obtained from the meta-analyses were then back-transformed, and the results were expressed as pooled proportions. The chi-square test was used to assess differences of the proportions between some of the predefined subgroups (p < 0.05, two sided), as reported below. Due to the large number of small studies with zero events, for the secondary outcome, the meta-analysis was performed using an approximated fixed-effect approach, weighting single study proportions for the study sample size.

The chi-square test was used to assess the statistical heterogeneity (with p < 0.1), which was quantified using the inconsistency index (I^2^). Heterogeneity was considered relevant when I^2^ > 50%. Stata software (version 16, StataCorp LLC) was used for the data analysis.

### Subgroup and sensitivity analyses

An important amount of heterogeneity was expected between the original studies, so prespecified subgroup analyses were performed in order to reduce the heterogeneity. The following factors were considered for subgroup analysis: different types of antiplatelet medication (aspirin, clopidogrel, prasugrel, ticagrelor, or DAPT); different ages considering the inclusion criteria of the original studies (< 60 years old vs. ≥ 60 years old); different outcome detection methods (follow-up vs. routine repeated CT scan).

Sensitivity analysis without the studies including moderate or severe TBI was eventually performed, considering two different definitions of mild TBI (TBI and GCS > 13 vs. GCS ≥ 13). Sensitivity analysis considering all patients lost at follow-up and all unexplained deaths as DB was also carried out.

## Results

### Study selection and characteristics

**Fig. 1:**
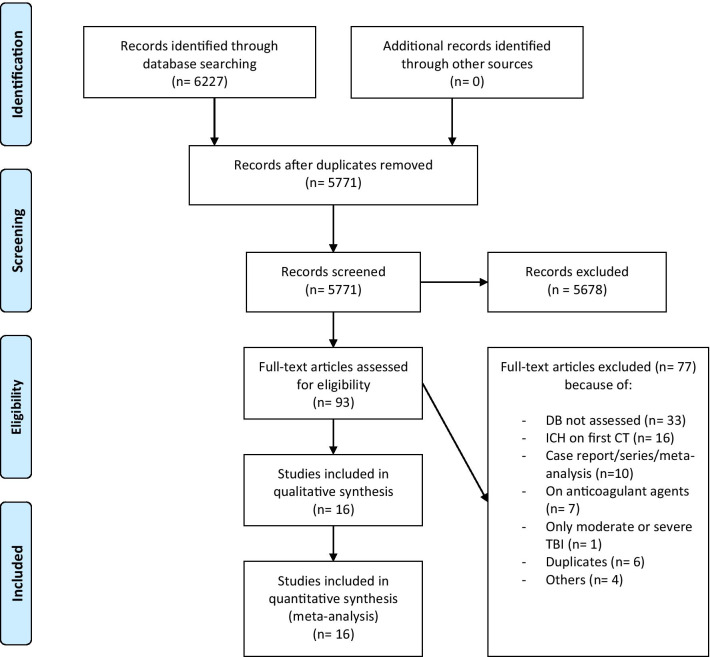
PRISMA 2009 flow diagram. DB = delayed bleeding; ICH = intracranial hemorrhage; CT = computed tomography; TBI = traumatic brain injury

A total of 6227 articles were identified from the database searches. ﻿After removing duplicates, 5771 articles remained, of which 5678 articles were excluded based on the title and abstract. The full texts of the remaining 93 articles were assessed for eligibility. After reading the full texts, 77 articles that did not meet our inclusion criteria were excluded. Sixteen studies, comprising 2930 patients, were finally included for qualitative and quantitative analysis (refer to Fig. 1 for details). Descriptive data are given for the entire population included in the primary studies.

The main characteristics of the selected studies are summarized in Table 1 and in the supplementary material (Additional file 2, Table A). The studies were published between 2009 and 2019; one study was performed in Italy [17], eleven studies in the USA [11, 18–27], three studies in Austria [28–30] and one in Australia [31]. Four studies were multicentric [20, 24, 27, 31], twelve had a retrospective design [11, 17–19, 22, 23, 25–28, 30, 31] and four had a prospective design [20, 21, 24, 29]. Two studies were described in abstracts only [18, 31], and the remaining 14 studies were published as full-text articles.

**Table 1:** Main characteristics of the original studies included in the meta-analysis

Study	Study design	Primary outcome of the study	Head trauma dynamic	Patients enrolled in the original study (No.)	Patients enrolled in this meta-analysis (No.)	Reference standard for delayed bleeding
ANTONI_2019	Retrospective	Incidence of delayed ICH after blunt head trauma in patients on antithrombotic agents	Blunt head trauma	793	108	RHCT after at least 24 h
BATTLE_2017	Retrospective	Incidence of delayed ICH in elderly trauma patients on antithrombotic agents	Blunt head trauma	110	44	RHCT at 6 h
CHENOWETH_2018	Prospective	Incidence of delayed ICH after head injury	Blunt head trauma	859	190	Telephone FU and EMR review
ERNSTBRUNNER_2016	Retrospective	S100B prediction for delayed ICH after MTBI in patients on LDA	MTBI (GCS > 13)	384	382	RHCT within 48 h
GALLIAZZO_2019	Retrospective	Incidence of ICH after MTBI in patients on antithrombotic agents	MTBI (GCS ≥ 13)	1846	131	RHCT at 24 h
GANETSKY_2017	Prospective	Incidence of ICH after minor falls in patients on antithrombotic agents	Head trauma after ground level fall	939	637	Informatic FU and EMR review at 30 days
HILL_2018	Retrospective	Incidence of delayed ICH after blunt head trauma in patients on antithrombotic agents	Blunt head trauma	338	213	RHCT within 48 h
HUANG_2019	Retrospective	Incidence of delayed ICH after blunt head trauma in patients on antithrombotic agents	Blunt head trauma	349	119	RHCT at 4–6 h
MANN_2018	Retrospective	Incidence of delayed ICH after a minor fall in elderly patients on antithrombotic agents	Head trauma after minor fall	218	114	RHCT before discharge
NISHIJIMA_2012	Prospective	Incidence of immediate and delayed ICH after blunt head trauma in patients on warfarin or clopidogrel	MTBI (GCS ≥ 13)	1064	239	Telephone FU and EMR review at 14 days
PECK_2011	Retrospective	Incidence of delayed ICH after blunt head trauma in patients on prescription antithrombotic agents	Blunt head trauma	500	103	EMR and informatic FU
SCANTLING_2017	Retrospective	Incidence of delayed ICH after MTBI in patients on antithrombotic agents	MTBI (GCS > 13)	234	165	RHCT at 12 h
STANITSAS_2016	Retrospective	Incidence of delayed ICH after blunt head trauma in patients on antithrombotic agents	Blunt head trauma	71	40	RHCT
SWAP_2016	Retrospective	Incidence of delayed ICH after blunt head trauma in patients on warfarin or clopidogrel	Blunt head trauma	491	260	FU from medical records at 60 days
TAUBER_2009	Prospective	Incidence of delayed ICH after MTBI in elderly patients on LDA	MTBI (GCS 15)	100	100	RHCT at 12–24 h
TAYLOR_2012	Retrospective	Incidence of delayed ICH after blunt head trauma in patients on antithrombotic agents	Blunt head trauma	159	85	RHCT within 48 h

GCS = Glasgow Coma Scale; MTBI = mild traumatic brain injury; LDA = low-dose acetylsalicylic acid; ICH = intracranial hemorrhage; RHCT = routine repeated head computed tomography scan; FU = follow-up; EMR = electronic medical record

*Only abstract available

Four studies enrolled only patients ≥ 65 years old [19, 23, 26, 29], one enrolled patients ≥ 60 years old [28], and one enrolled patients ≥ 55 years old [20]. The others included adult patients without age restriction [11, 17, 18, 21, 24, 25, 30] or did not specify any age restrictions in the inclusion or exclusion criteria [22, 27, 31].

Five studies enrolled only patients with MTBI [17, 24, 26, 28, 29]; the definition of MTBI was not uniform across these five studies, and it was not always clarified. However, there was a consensus on the definition of MTBI as GCS ≥ 13. The severity of trauma and the GCS of patients were not clearly specified in the inclusion criteria of 11 studies, although most of the studies enrolled in the end only patients with MTBI. Only two out of eleven studies specified a minor fall or a minor dynamic as the cause of the trauma [21, 23], while the remaining nine studies enrolled patients with a more generical blunt head injury.

As far as outcome detection is concerned, eleven studies used a routine repeated head CT scan (within 6–48 h) to reveal DB; while in the remaining five studies, CT was repeated only if clinically indicated during the follow-up of various durations, from 14 to 60 days following the index event [20, 21, 24, 25, 27].

The first CT scan was performed by protocol at arrival in all of the included studies. Only one study specified a defined time interval between arrival and the first CT scan [28], and only one other study specified the mean time interval between arrival and the first CT scan [29]. The time to the repeated CT scan was quite variable among studies. A routine repeated head CT scan was performed at 6 h after the first CT scan in three studies [11, 19, 25], while a 12-h interval was reported in two studies [26, 29]. The repeated CT scan was performed at 24 h after the first CT scan in two studies [17, 30] and within 48 h in three studies [22, 28, 31]; only one study—an only abstract publication—did not specify the time lag between the two head CT scans [18].

Considering the different antiplatelet agents (APAs) used in the various studies, two studies included only patients administered acetylsalicylic acid (ASA) [28, 29], three studies included only patients on at least one prescription APA [19, 25, 26], and all of the other studies included patients on ASA, clopidogrel, or DAPT. Data on other P2Y receptor inhibitors were very scant and incomplete. Only a few patients took dipyridamole alone or in combination with ASA.

### Study outcomes

**Fig. 2:**
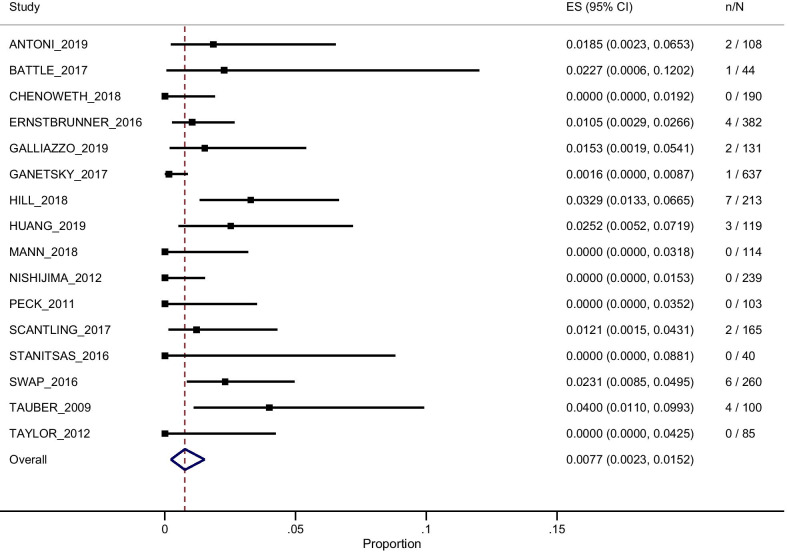
Random-effects meta-analysis results for the primary outcome, i.e., delayed bleeding. ES (95% CI) = estimated mean value and 95% confidence interval; n/N = number of events/number of patients

The pooled absolute risk for DB was 0.77% (95% CI 0.23–1.52%), ranging from 0% in some studies [18, 20, 23–25, 31] to 4% in the study by Tauber et al. (see Fig. 2 for details).

The secondary outcome chosen for the meta-analysis was the absolute risk of clinically relevant bleeding. The absolute risk of DB was calculated with a fixed-effect meta-analysis. Thirteen studies reported zero events, while the remaining three studies reported five cases of bleeding. The pooled absolute risk of clinically relevant bleeding was 0.17% (95% CI 0.06–0.40%) (see Table 2 for details).

**Table 2:** Fixed-effects meta-analysis results for clinically relevant delayed bleeding

Antiplatelet agent subgroup	No. of enrolled studies	No. of events due to DB (deaths or neurosurgery)	No. of patients at risk	CRDB mean estimated risk (95% CI)
All	16	5	2930	0.17% (0.06–0.40%)
ASA	11	3	1706	0.18% (0.04–0.51%)
Clopidogrel	9	0	265	0.00% (0.00–1.38%)
DAPT	6	0	175	0.00% (0.00–2.09%)

CRDB = clinically relevant delayed bleeding as defined in the text; 95% CI = 95% confidence interval; All = all patients included in the analysis; ASA = acetylsalicylic acid; DAPT = dual antiplatelet therapy

### Risk of bias assessment

According to the adapted version of QUADAS-2 [16], the applicability to the review question was good for most of the selected studies. Some studies had an unclear concern for applicability because the rate of patients afflicted by non-mild TBI was not specified in the original studies [18–21, 23, 31].

Only 4 of the 16 studies were rated as having a low risk of bias [25, 26, 29, 30]. In addition, four studies were rated as having an unclear risk of bias [23, 27, 28, 31] because there was insufficient transparency in the patient flow and timing. All of the other studies were rated as having a high risk of bias in at least one section of the adapted QUADAS-2 risk of bias assessment tool. Most of them were judged as having a high risk of bias due to possible systematic errors in the patient flow and timing, while some of them were judged as having a high risk of bias because of inappropriate application of the reference standard (i.e., follow-up or routine repeated head CT scan).

**Fig. 3:**
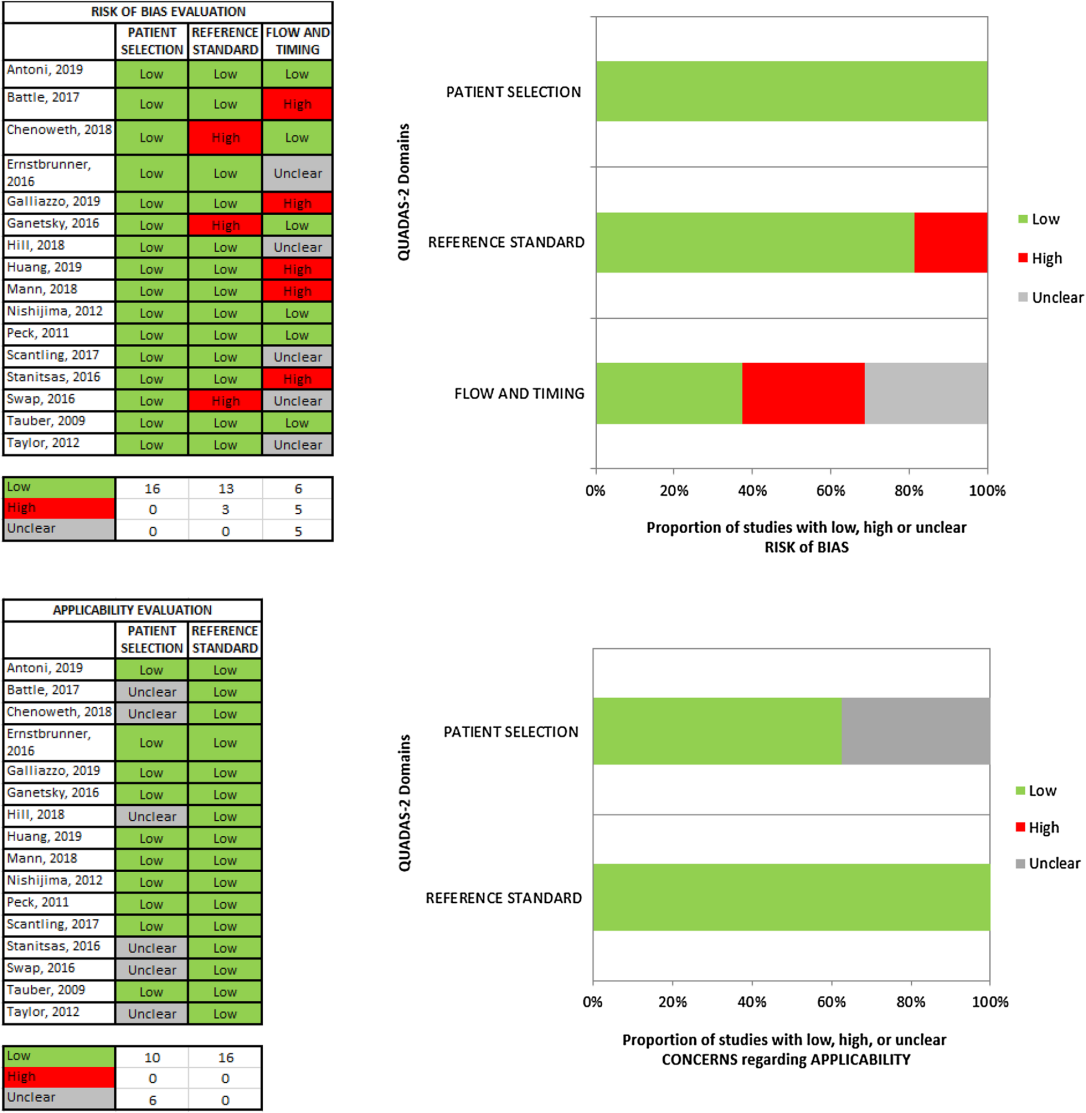
Risk of bias and concern about applicability to the review question among the original studies. See the color legend within the figure defining the risk of bias and the concern about applicability to the review question

No risk for systematic errors in patient selection was found. All the details on the risk of bias assessment are shown in Fig. 3 and in the supplementary material (Additional file 1).

### Heterogeneity and subgroup analysis

Across the included studies, a substantial heterogeneity for the primary outcome was revealed (p = 0.0007; I^2^ = 61%). Heterogeneity among studies is described in detail in the supplementary material.

Subgroup analyses were performed to reduce the heterogeneity and to evaluate the bleeding risk associated with different types of APAs (aspirin or clopidogrel) and with DAPT. Additionally, to further explore and reduce the heterogeneity, the subgroups were stratified by the outcome detection method (follow-up or CT scan) and by age, as specified in the inclusion criteria of each original study.

**Fig. 4:**
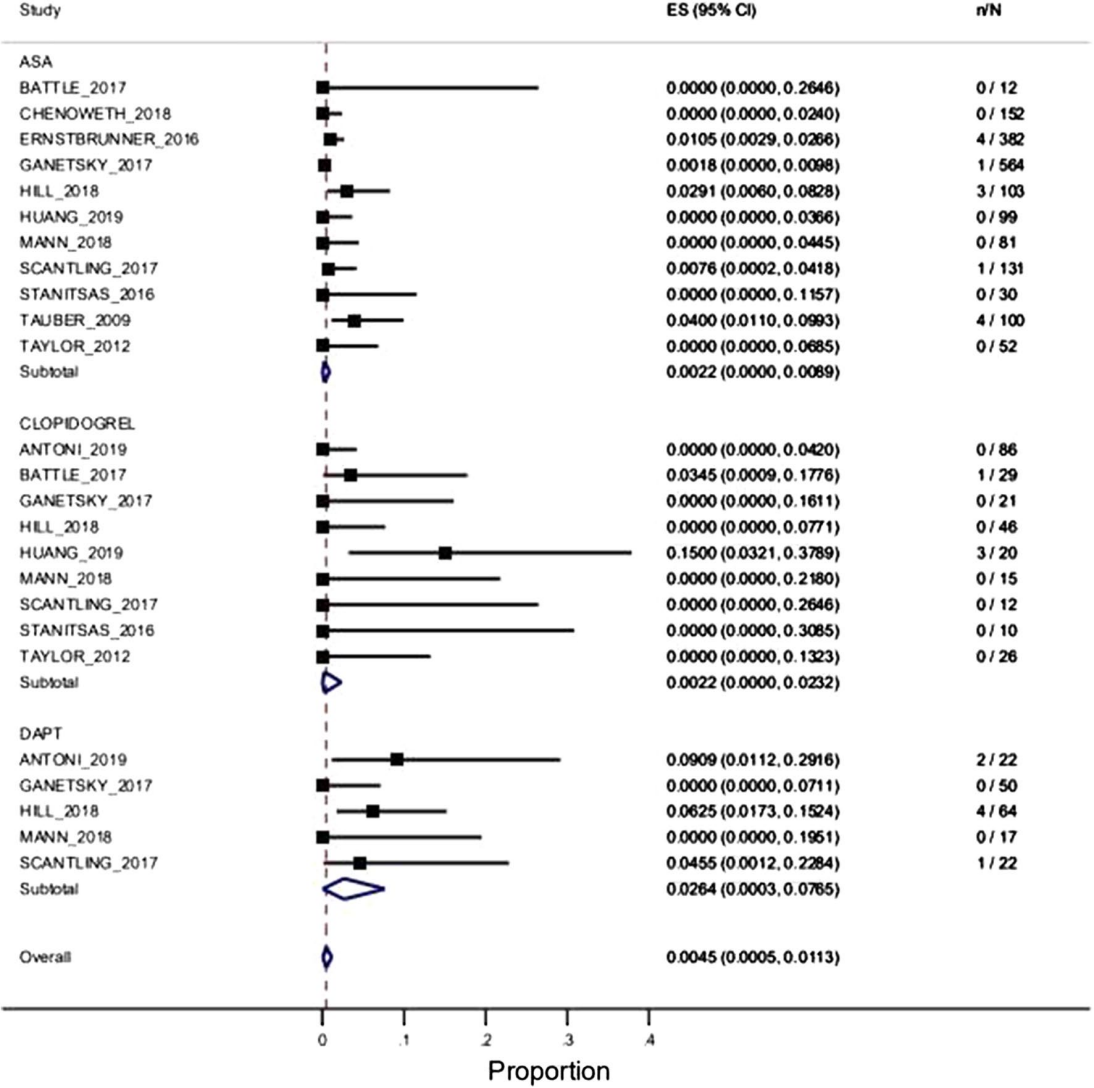
Random-effects meta-analysis results for the primary outcome according to different antiplatelet agent subgroups (acetylsalicylic acid, clopidogrel, and double antiplatelet therapy). ES (95% CI): estimated mean value and 95% confidence interval; n/N: number of events/number of patients

According to different APAs, the risk of DB was 0.22% (95% CI 0.0–0.89%) for ASA [11 studies and 1706 enrolled patients], 0.22% (95% CI 0.0–2.32%) for clopidogrel [9 studies and 265 enrolled patients], and 2.64% (95% CI 0.03–7.65%) for DAPT [5 studies and 175 enrolled patients], as presented in Fig. 4 and in the supplementary material (Additional file 2, Table B). The incidence of DB was significantly higher in the DAPT patients compared to the ASA patients (2.64% vs. 0.22%; p = 0.04), while the difference was not significant compared to the clopidogrel patients (0.22% vs. 2.64% p = 0.28). A significant difference in DB between the ASA and clopidogrel groups (0.22% vs. 0.22% p = 0.31) was not found.

Considering the patient age, the studies were divided into two subgroups: studies enrolling patients of all ages and patients enrolling only patients > 60 years old. The risk of DB was 1.09% (95% CI 0.18–2.52%) in studies enrolling elderly patients [5 studies and 805 enrolled patients], and it was 0.64% (95% CI 0.07–1.57%) in studies without any age restriction [11 studies and 2125 enrolled patients]; the results are shown in Table 3 and in the supplementary material (Additional file 3, Figure A). In an additional analysis, the included studies were further divided into two subgroups considering an age cut-off of 65 years old: the risk of DB was 1.27% (95% CI 0.02–3.68%) in studies enrolling patients older than 65 years old [4 studies and 423 enrolled patients], whereas it was 0.67% (95% CI 0.13–1.48%) in studies without any age restriction [11 studies and 2507 enrolled patients]. Only a slight reduction in primary outcome heterogeneity was observed in the subgroup of original studies enrolling patients older than 65 years (I^2^ = 53%). For details, see Figure B in Additional file 3.

**Table 3:** Subgroup analysis results for the primary outcome considering variability in outcome assessment and age

Study	Repeated CT scan	Follow-up	Age ≥ 60 yo	Age < 60 yo
ANTONI_2019	1.85% (0.23–6.53%)	NA	NA	1.85% (0.23–6.53%)
BATTLE_2017	2.27% (0.06–12.02%)	NA	2.27% (0.06–12.02%)	NA
CHENOWETH_2018	NA	0.00% (0.00–1.92%)	NA	0.00% (0.00–1.92%)
ERNSTBRUNNER_2016	1.05% (0.29–2.66%)	NA	1.05% (0.29–2.66%)	NA
GALLIAZZO_2019	1.53% (0.19–5.41%)	NA	NA	1.53% (0.19–5.41%)
GANETSKY_2017	NA	0.16% (0.00–0.87%)	NA	0.16% (0.00–0.87%)
HILL_2018	3.29% (1.33–6.65%)	NA	NA	3.29% (1.33–6.65%)
HUANG_2019	2.52% (0.52–7.19%)	NA	NA	2.52% (0.52–7.19%)
MANN_2018	0.00% (0.00–3.18%)	NA	0.00% (0.00–3.18%)	NA
NISHIJIMA_2012	NA	0.00% (0.00–1.53%)	NA	0.00% (0.00–1.53%)
PECK_2011	NA	0.00% (0.00–3.52%)	NA	0.0% (0.00–3.52%)
SCANTLING_2017	1.21% (0.15–4.28%)	NA	1.21% (0.15–4.31%)	NA
STANITSAS_2016	0.00% (0.00–8.81%)	NA	NA	0.05 (0.00–8.81%)
SWAP_2016	NA	2.31% (0.85–4.95%)	NA	2.31% (0.85–4.95%)
TAUBER_2009	4.00% (1.10–9.93%)	NA	4.0% (1.10–9.93%)	NA
TAYLOR_2012	0.00% (0.00–4.25%)	NA	NA	0.00% (0.00–4.25%)
No. studies	11	5	5	11
DB mean estimated risk	1.29% (0.66–2.18%)	0.22% (0.00–1.05%)	1.09% (0.18–2.52%)	0.64% (0.07–1.57%)
p value for heterogeneity	p = 0.2011	p = 0.0207	p = 0.1491	p = 0.0008
I^2^ statistic	26%	66%	41%	67%

The results are presented as the mean estimates of delayed bleeding risk and 95% confidence intervals (in parentheses). Repeated CT scan: delayed bleeding detection by means of a routine repeated CT scan; Follow-up = event detection by means of a clinical follow-up; Age ≥ 60 yo = only studies enrolling patients older than 60 years old are included; Age < 60 yo = only studies enrolling patients younger than 60 years old are included; No. studies = number of the studies included in the analysis; NA = not assessed in the corresponding original study

As far as different outcome detection methods are concerned, the risk of DB among studies that repeated the CT scan by protocol [11 studies and 1501 enrolled patients] was 1.29% (95% CI 0.6–2.18%), compared to 0.22% (95% CI 0.00–1.05%) for studies that detected DB by means of a clinical follow-up [5 studies and 1429 enrolled patients]; the details are shown in Table 3 and in the supplementary material (Additional file 3, Figure C). Heterogeneity was substantially reduced among the studies that used only routine repeated head CT as the reference standard for DB (I^2^ = 26%).

### Sensitivity analysis

Sensitivity analysis was performed considering all of the patients lost at follow-up and all deaths detected at follow-up as events (i.e., DB). According to this sensitivity analysis, the pooled risk of DB was 1.70% (95% CI 0.93–2.67%); see Table 4 and the supplementary material for details (Additional file 3, Figure D).

**Table 4:** Delayed bleeding risk according to the two different sensitivity analyses performed

**Study**	**MTBI**	**Sensitivity**
ANTONI_2019	NA	1.85% (0.23–6.53%)
BATTLE_2017	NA	2.27% (0.06–12.02%)
CHENOWETH_2018	NA	7.11% (3.94–11.64%)
ERNSTBRUNNER_2016	1.05% (0.29–2.66%)	1.05% (0.29–2.66%)
GALLIAZZO_2019	1.53% (0.19–5.41%)	1.53% (0.19–5.41%)
GANETSKY_2017	NA	3.61% (2.30–5.37%)
HILL_2018	NA	3.29% (1.33–6.65%)
HUANG_2019	NA	2.52% (0.52–7.19%)
MANN_2018	NA	0.00% (0.00–3.18%)
NISHIJIMA_2012	0.00% (0.00–1.53%)	1.23% (0.26–3.57%)
PECK_2011	NA	0.00% (0.00–3.52%)
SCANTLING_2017	1.21% (0.15–4.31%)	1.2% (0.15–4.28%)
STANITSAS_2016	NA	0.00% (0.00–8.81%)
SWAP_2016	NA	2.31% (0.85–4.95%)
TAUBER_2009	4.00% (1.10–9.93%)	4.00% (1.10–9.93%)
TAYLOR_2012	NA	0.00% (0.00–4.25%)
No. studies	5	16
DB mean estimated risk	1.04% (0.15–2.49%)	1.70% (0.93–2.67%)
p value for heterogeneity	p = 0.0334	p = 0.0012
I^2^ statistic	62%	60%

The results are presented as the mean estimates of delayed bleeding risk and 95% confidence intervals (in parentheses). GCS = Glasgow Coma Scale; MTBI = mild traumatic brain injury: includes only studies enrolling patients with TBI and a GCS ≥ 13; Sensitivity = includes all the studies considering unexplained deaths and patients lost at follow-up as events (i.e. delayed bleeding); No. studies = number of the studies included in the analysis; NA = not assessed in the corresponding original study

Sensitivity analysis including only studies enrolling MTBI patients considering two different definitions of MTBI (TBI and GCS > 13 vs. GCS ≥ 13) was also performed. If studies enrolling only patients presenting with a TBI and GCS ≥ 13 [5 studies and 1017 enrolled patients] were considered, the incidence of DB was 1.04% (95% CI 0.15–2.49%); if studies enrolling only patients presenting with a TBI and GCS > 13 [3 studies and 647 enrolled patients] were considered, the incidence of DB was 1.55% (95% CI 0.39–3.30%); for details, refer to Table 4 and Figure E in Additional file 3 [17, 24, 26, 28, 29].

## Discussion

The main findings of this systematic review and meta-analysis were as follows: i) There is a low risk of DB in MTBI patients administered APAs; ii) There is an even lower risk of clinically relevant DB among these patients; iii) A routine repeated CT scan detects more DB compared to a clinical follow-up assessment only, but most of these bleedings are not associated with major events such as neurosurgical intervention or death; and iv) DAPT patients are at a higher risk of DB compared to ASA-only patients.

We aimed to perform this systematic review and meta-analysis to quantify the risk of DB after MTBI in patients on antiplatelet therapy in order to compare the possible diagnostic yield of a repeated head CT or a short-term clinical follow-up to a single head CT scan in MTBI patients administered APAs.

We found that the risk of DB in MTBI patients on antiplatelet therapy was as low as 0.77% (95% CI 0.2–1.5%) and that the absolute risk of clinically relevant DB was 0.17% (95% CI 0.06–0.4%). Furthermore, our review showed that a clinical follow-up is far less sensitive at detecting DB compared to a routine repeated head CT scan (follow-up: 0.22%, 95% CI 0.00–1.05 vs. routine repeated CT scan: 1.29%, 95% CI 0.6–2.18%.). We are aware that a repeated CT scan is the reference standard for DB detection after MTBI; however, in everyday clinical practice, waiting for a repeated CT scan exposes the patient to the risks of a prolonged ED stay, often without any relevant clinical benefit. Our review shows that even if the CT scan detects more DB when compared to an observation-only strategy, it seldom leads to major events such as neurosurgical intervention or death. Indeed, we found that the risk of clinically relevant DB is very similar to the risk of DB when assessed through a clinical follow-up. We speculate that a routine repeated head CT scan could reveal more minor DB than a clinical observation or follow-up, without leading to any relevant change in patient management.

When considering guideline recommendations for CT scan execution in MTBI patients on antiplatelet therapy, the topic is only covered by the Scandinavian and NICE guidelines [32, 33]. The former only recommends a first head CT (or in-hospital observation ≥ 12 h after injury) for patients ≥ 65 years and on antiplatelet medication, while imaging is not required for younger subjects. The National Institute for Health and Care Excellence guidelines of the UK report that scientific data are insufficient to provide recommendations on the management of these patients. A recent meta-analysis has demonstrated a small increased risk of immediate ICH in MTBI patients on antiplatelet therapy, especially if concomitant with another risk factor for ICH such as GCS < 15 or age > 65 years [34]. No guideline recommendations exist for routine repeated CT scans in MTBI patients on antiplatelet therapy, since the literature on this topic is very scant. We think that the results of our work could be useful in case of MTBI guideline revisions. The most valuable results that should be included in future guideline revisions are as follows: the low overall risk of DB in patients with MTBI and administered APAs, the low risk of DB in patients with a routine repeated head CT scan, and patients on DAPT seem to be at a higher risk of DB compared to patients taking only ASA.

Only one recent meta-analysis has analyzed the risk of DB in MTBI patients on APAs [11]. Huang and colleagues have shown that in MTBI patients on antithrombotic therapy, repeat scans should be discretionarily based on neurologic assessments and that routine repeated CT may identify a larger proportion of minor delayed ICH. In addition, they found a slightly higher risk of DB compared to our results. Their meta-analysis included all types of antithrombotic agents, but a pooled estimate of DB for APAs only is not available. Even when considering the only comparable subgroup—ASA-only patients—we believe that the findings are not completely comparable to our work. The pooled results reported by Huang and colleagues included both APAs and anticoagulant agents, and the meta-analysis lacks some of the original studies that are included in our work [17, 18, 21, 30]. Moreover, their meta-analysis, even the “ASA-only” patient subgroup, did not include any study that used a clinical follow-up as the primary outcome assessment. Furthermore, the work by Huang et al. does not show any data about DB and DAPT. In regard to DB risk according to different antiplatelet therapy subgroups, our work highlights that while DB seems to be comparable between patients taking clopidogrel vs. ASA, it is far more elevated in patients on DAPT. Due to the elevated risk of DB in MTBI patients on DAPT, we suggest a case-by-case evaluation of the need for a second CT scan (at least 6 h after the first CT scan) before discharge in this subgroup of patients in order not to miss any clinically relevant DB. Of note, in the original studies included in our systematic review, there were very few data on newer APAs, such as ticagrelor or prasugrel, which do not allow any further consideration on newer antiplatelet agents.

Substantial heterogeneity was present in the risk of DB according to the original studies. In fact, DB risk for MTBI patients on APAs ranges from 0% in some studies [18, 20, 23–25, 31] to 4% in the study by Tauber et al. [29]. Even if we tried to analyze and reduce the heterogeneity through prespecified subgroup analyses, many precautions still must be taken before our findings can be generalized to every MTBI patient on antiplatelet therapy (refer to Tables 3, 4 and to table B in Additional file 2 for details on heterogeneity).

Our systematic review has some limitations that must be addressed. First, the original studies were very biased, so our data should be interpreted with caution. We rated most of the original studies as “low quality.” Most of the biases were detected in the “flow and timing” section of the adapted QUADAS-2 quality assessment tool. Indeed, most of the studies had a retrospective design; thus, a common systematic error was to not report the MTBI patients with a negative first CT scan who did not undergo a repeated head CT scan before discharge in the flow diagram of the original study [11, 17–20, 23, 24, 27, 28, 31]. It is likely that this bias could have increased the proportion of patients with DB in the original studies, since those patients who were deemed to be at low risk did not have their second CT scan performed due to medical decisions. Nevertheless, retrospective studies enroll patients according to local MTBI management protocols, and they are closer to everyday clinical practice. Second, another common source of bias was the way of outcome assessment by means of a clinical follow-up: some of the original studies had a loose or poor quality follow-up, and some had lost patients at follow-up [19, 21, 22,]. To face this issue, we performed a sensitivity analysis considering all patients lost at follow-up or all unexplained deaths as events. Even in this worst-case scenario, we detected a mean DB incidence as low as 1.7% (95% CI 0.93–2.67%). Third, some studies had an unclear concern for applicability to the review question because the rate of patients with non-mild TBI was not specified in the original studies [18–21, 23, 31]. Indeed, we preferred to increase the sensitivity and overestimate the rate of DB rather than underestimate it; therefore, some non-mild TBI patients were enrolled.

## Conclusions

Our systematic review showed a very low risk of DB in MTBI patients on antiplatelet therapy. We believe that such a low rate of DB could not justify routine repeated CT scans in MTBI patients administered a single APA. We speculate that in the case of clinically stable patients, a repeated head CT scan could be useful for select high-risk patients and for patients on DAPT before discharge.

## Supplementary Information

**Additional file 1.** Adapted QUADAS-2 template, used for risk of BIAS assessment.

**Additional file 2.** Additional data about the general characteristcs of the included studies (Table A) and about the subgroup analysis performed if considering variability in the administered antiplatelet agents (Table B).

**Additional file 3.** Forest plots about the subgroup analysis performed if considering variability in age and in the means of delayed bleeding detection (Figure A to C), and about the two sensitivity analyses performed (Figure D and E).

## Data Availability

All data and materials are available from the corresponding author upon request.

## Abbreviations

DB: Delayed intracranial bleeding
MTBI: Mild traumatic brain injury
GCS: Glasgow Coma Scale
APA: Antiplatelet agent
CT: Computed tomography
DAPT: Dual antiplatelet therapy
TBI: Traumatic brain injury
ED: Emergency department
ICH: Intracerebral hemorrhage
PRISMA: Preferred Reporting Items for Systematic Reviews and Meta-Analyses
QUADAS-2: Quality Assessment of Diagnostic Accuracy study-2
CI: Confidence interval
ASA: Acetylsalicylic acid
