# Supplementary material for: Incidence of delayed bleeding in patients on antiplatelet therapy after mild traumatic brain injury: a systematic review and meta-analysis
Source: Scand J Trauma Resusc Emerg Med. 2021 Aug 23;29:123. doi: 10.1186/s13049-021-00936-9 (PMC8381571; doi:10.1186/s13049-021-00936-9)
Supplement: Supplementary file 2 — Additional file 2. Additional data about the general characteristcs of the included studies (Table A) and about the subgroup analysis performed if considering variability in the administered antiplatelet agents (Table B). [file 13049_2021_936_MOESM2_ESM.pdf]

**Antoni A, 2019**	**Country**	Austria
	**Single/multicenter**	Single center
	**Study design**	Retrospective
	**Mild TBI definition**	NA
	**Primary outcome**	Incidence of delayed ICH
	**Inclusion criteria**	Patients ≥18 years old; blunt head trauma; ongoing antithrombotic therapy; no pathologies in their initial CT
	**Exclusion criteria**	NA
	**Recruitment time**	January 2012 - April 2014
	**Patients enrolled**	793
	**Mean Age, years**	81(range 32–102)
	**Male (%)**	340 (42.9)
	**Time from trauma to ED presentation**	NA
	**Time from first to second CT scan**	Minimum observation time of 24 h
	**Patients enrolled in our meta-analysis**	108
	**Reference standard for DB**	RHCT after at least 24 h
	**Aspirin**	0
	**Clopidogrel**	86
	**Other antiplatelet**	0
	**Dual antiplatelet**	22
**Battle B, 2017**	**Country**	USA
	**Single/multicenter**	Single center
	**Study design**	Retrospective
	**Mild TBI definition**	NA
	**Primary outcome**	Utility of 6-hour repeat head CT among elderly trauma patients across a broad spectrum of anticoagulation therapies with negative initial imaging
	**Inclusion criteria**	Patients > 65 years old; head trauma; anticoagulant or antiplatelet therapy; initially negative non-contrast head CT followed by a repeat examination at 6 hours.
	**Exclusion criteria**	Hemorrhage on the initial head CT; follow-up scans that exceeded the 6-hour mark by more than 2 hours; unknown type of anticoagulant/antiplatelet therapy.
	**Recruitment time**	July 2015 - April 2016
	**Patients enrolled**	110
	**Mean Age, years (mean±SD, or median (IQR))**	>65 years (not specified)
	**Male (%)**	NA
	**Time from trauma to ED presentation**	NA
	**Time from first to second CT scan**	6 hours
	**Patients enrolled in our meta-analysis**	44
	**Reference standard for DB**	6h RHCT
	**Aspirin**	12
	**Clopidogrel**	29
	**Other antiplatelet**	3
	**Dual antiplatelet**	0
**Chenoweth J, 2018**	**Country**	USA
	**Single/multicenter**	Multicentric
	**Study design**	Prospective observational cohort study
	**Mild TBI definition**	Isolated head injury was defined as an Abbreviated Injury Scale score less than 3 in all non-head body regions

	**Primary outcome**	Incidence of delayed traumatic ICH on cranial CT within 14 days of the index ED visit in the study without the patient having experienced an additional head injury
	**Inclusion criteria**	Patients ≥ 55 years old; head trauma transported to a participating hospital by EMS
	**Exclusion criteria**	Penetrating head trauma; patients with interfacility transfers; ICH on the initial cranial CT; no cranial CT at their index ED visit; declined consent for a follow-up telephone call; patients who were without reliable means for such a call; incarcerated people
	**Recruitment time**	August 2015 – September 2016
	**Patients enrolled**	859
	**Mean Age, years (median (IQR))**	75 (64-85)
	**Male (%)**	389 (45.3)
	**Time from trauma to ED presentation**	NA
	**Time from first to second CT scan**	NA
	**Patients enrolled in our meta-analysis**	190
	**Reference standard for DB**	Telephone FU 14 days after discharge and EMR review
	**Aspirin**	152
	**Clopidogrel**	NA
	**Other antiplatelet**	38
	**Dual antiplatelet**	0
**Ernstbrunner L, 2016**	**Country**	Austria
	**Single/multicenter**	Single center
	**Study design**	Retrospective
	**Mild TBI definition**	GCS of 14–15
	**Primary outcome**	To analyze whether secondary ICH after mild head injury in patients ≥ 60 years old with LDA prophylaxis at the time of admission can reliably be predicted by the serum level of S100B
	**Inclusion criteria**	≥ 60 years of age; daily LDA prophylaxis (50–100 mg); isolated mild head injury with a GCS of 14–15; negative head CT scan within 3 hours; no hypertensive irregularities during the in-hospital observation period (systolic blood pressure <150mm Hg)
	**Exclusion criteria**	Patients taking anticoagulants such as heparin, warfarin, coumarin, clopidogrel or nonsteroidal anti-inflammatory drugs; hematological or oncological diseases; moderate or severe head injuries
	**Recruitment time**	November 2008 - May 2012
	**Patients enrolled**	384
	**Mean Age, years (mean ± SD)**	81.8±8.9
	**Male (%)**	157 (41)
	**Time from trauma to ED presentation**	< 3 hours
	**Time from first to second CT scan**	Within 48 hours<br>21.1 (±6.8) hours in patients with no ICH<br>21.5 (±3.7) hours in patients with ICH
	**Patients enrolled in our meta-analysis**	382
	**Reference standard for DB**	RHCT within 48 h
	**Aspirin**	382
	**Clopidogrel**	0
	**Other antiplatelet**	0
	**Dual antiplatelet**	0

**Galliazzo S, 2019**	**Country**	Italy
	**Single/multicenter**	Single center
	**Study design**	Retrospective
	**Mild TBI definition**	GCS 13-15
	**Primary outcome**	ICH after mild traumatic brain injury with a GCS ≥13 in patients treated with different antithrombotic therapy
	**Inclusion criteria**	Patients > 18 years old; traumatic brain injury; GCS 13 to 15
	**Exclusion criteria**	Any regimen of low molecular weight heparin
	**Recruitment time**	January 2015 – September 2017
	**Patients enrolled**	1846
	**Mean Age, years (median (IQR))**	71 (46–83)
	**Male (%)**	926 (50)
	**Time from trauma to ED presentation**	NA
	**Time from first to second CT scan**	Between 6 hours and 24 hours
	**Patients enrolled in our meta-analysis**	131
	**Reference standard for DB**	24h RHCT
	**Aspirin**	NA
	**Clopidogrel**	NA
	**Other antiplatelet**	NA
	**Dual antiplatelet**	NA
**Ganetsky M, 2017**	**Country**	USA
	**Single/multicenter**	Single center
	**Study design**	Prospective observational cohort study
	**Mild TBI definition**	NA
	**Primary outcome**	Rates of ICH in ED patients on anticoagulants or antiplatelet agents who sustain a ground-level fall
	**Inclusion criteria**	Consecutive adult patients (>18 years of age); ground-level fall or lesser mechanism of injury; CT head performed; on aspirin, clopidogrel, prasugrel, ticagrelor, warfarin, dabigatran, rivaroxaban, or enoxaparin
	**Exclusion criteria**	Therapy with edoxaban (not received FDA approval at the initiation of this study); patients transferred from an outside hospital with identified injuries or an injury that occurred > 24 hours prior to presentation
	**Recruitment time**	June 2013 - November 2015
	**Patients enrolled**	939
	**Mean Age, years (mean ± SD)**	78.3 ±11.9
	**Male (%)**	419 (44.6)
	**Time from trauma to ED presentation**	NA
	**Time from first to second CT scan**	NA
	**Patients enrolled in our meta-analysis**	637
	**Reference standard for DB**	30 days informatic FU
	**Aspirin**	564
	**Clopidogrel**	21
	**Other antiplatelet**	0
	**Dual antiplatelet**	52
**Hill JH, 2018**	**Country**	USA
	**Single/multicenter**	Single center
	**Study design**	Retrospective
	**Mild TBI definition**	NA

	**Primary outcome**	Incidence of delayed ICH on follow-up CT exam
	**Inclusion criteria**	Patients who had blunt head trauma; on an anticoagulant/antiplatelet therapy; initial head CT negative for ICH; follow-up head CT completed within 48 hours of the initial CT
	**Exclusion criteria**	Patients not on anticoagulant or anti-platelets therapy; penetrating injury; initial head CT positive for ICH; no follow-up exam within 48 hours after the initial CT
	**Recruitment time**	January 2008 - December 2012
	**Patients enrolled**	338
	**Mean Age, years (mean±SD)**	69.3±14.47
	**Male (%)**	195 (57.7)
	**Time from trauma to ED presentation**	NA
	**Time from first to second CT scan**	Within 48 hours
	**Patients enrolled in our meta-analysis**	213
	**Reference standard for DB**	RHCT within 48 h
	**Aspirin**	103
	**Clopidogrel**	46
	**Other antiplatelet**	NA
	**Dual antiplatelet**	64
**Huang G, 2019**	**Country**	USA
	**Single/multicenter**	Single center
	**Study design**	Retrospective
	**Mild TBI definition**	A documented statement that the patient hit his/her head; any craniofacial soft tissue injury, cephalohematoma, craniofacial fracture, or cognitive alteration
	**Primary outcome**	Incidence of delayed traumatic ICH in patients with blunt trauma to the head taking pre-injury antithrombotic agents
	**Inclusion criteria**	Patients ≥18 years old; sustained blunt head trauma; taking any antithrombotic agent; initially negative head CT
	**Exclusion criteria**	Patients < 18 years old; initially positive head CT; not taking an antithrombotic agent
	**Recruitment time**	July 2014 - December 2015
	**Patients enrolled**	349
	**Mean Age, years**	74.7
	**Male (%)**	159 (46)
	**Time from trauma to ED presentation**	NA
	**Time from first to second CT scan**	within 4-6 h
	**Patients enrolled in our meta-analysis**	119
	**Reference standard for DB**	4-6h RHCT
	**Aspirin**	99
	**Clopidogrel**	20
	**Other antiplatelet**	0
	**Dual antiplatelet**	NA
**Mann N, 2018**	**Country**	USA
	**Single/multicenter**	Single center
	**Study design**	Retrospective
	**Mild TBI definition**	Minor fall as defined by the National Trauma Data Standard as a fall from less than 10 ft

	**Primary outcome**	Frequency of delayed ICH in an elderly cohort presenting following a minor fall while taking pre-injury anticoagulant or antiplatelet therapy
	**Inclusion criteria**	Patients ≥65 years old; admission to the trauma service, sustaining a minor fall as defined by the National Trauma Data Standard as a fall from less than 10 ft; having a documented head CT scan in the trauma registry; taking an anticoagulant or antiplatelet therapy; an initial and routine repeat head CT performed prior to discharge
	**Exclusion criteria**	NA
	**Recruitment time**	January 2014 - December 2015
	**Patients enrolled**	218
	**Mean Age, years (mean±SD)**	81.6±7.65
	**Male (%)**	91 (41.74)
	**Time from trauma to ED presentation**	Within 6 hours
	**Time from first to second CT scan**	NA
	**Patients enrolled in our meta-analysis**	114
	**Reference standard for DB**	RHCT before discharge
	**Aspirin**	81
	**Clopidogrel**	15
	**Other antiplatelet**	1
	**Dual antiplatelet**	17
**Nishijima D, 2012**	**Country**	USA
	**Single/multicenter**	Multicentric
	**Study design**	Prospective observational study
	**Mild TBI definition**	Any blunt head injury regardless of loss of consciousness or amnesia
	**Primary outcome**	Prevalence and incidence of immediate and delayed traumatic ICH in patients with blunt head trauma who were receiving either warfarin or clopidogrel
	**Inclusion criteria**	Adult (aged ≥18 years) ED patients; blunt head trauma; preinjury warfarin or clopidogrel use (within the previous 7 days)
	**Exclusion criteria**	patients with known injuries who were transferred from outside facilities because their inclusion would falsely inflate the prevalence of traumatic ICH; patients with concomitant warfarin and clopidogrel use
	**Recruitment time**	April 2009 - January 2011
	**Patients enrolled**	1064
	**Mean Age, years (mean±SD)**	75.4±12.7
	**Male (%)**	502 (47.1)
	**Time from trauma to ED presentation**	NA
	**Time from first to second CT scan**	NA
	**Patients enrolled in our meta-analysis**	239
	**Reference standard for DB**	telephone FU 14 days after discharge and EMR review
	**Aspirin**	0
	**Clopidogrel**	ND
	**Other antiplatelet**	0
	**Dual antiplatelet**	ND
**Peck KA, 2011**	**Country**	USA
	**Single/multicenter**	Single center
	**Study design**	Retrospective

	**Mild TBI definition**	NA
	**Primary outcome**	Incidence of delayed ICH
	**Inclusion criteria**	Age ≥15 years; blunt mechanism of injury; preinjury use of an anticoagulant or antiplatelet agent (warfarin, clopidogrel, heparin, enoxaparin, or dipyridamole and aspirin in combination. Patients with a concurrent history of aspirin use were not excluded, but aspirin use alone was not sufficient for inclusion in the study
	**Exclusion criteria**	Warfarin as the sole inclusion agent used and the admission international normalized ratio was within normal range (< 1.3)
	**Recruitment time**	January 2006 – August 2009
	**Patients enrolled**	500
	**Mean Age, years (mean±SD)**	75.0 ± 13.6
	**Male (%)**	210 (49.5)
	**Time from trauma to ED presentation**	NA
	**Time from first to second CT scan**	8.8 ± 10.2 hours
	**Patients enrolled in our meta-analysis**	103
	**Reference standard for DB**	EMR review and informatic FU
	**Aspirin**	0
	**Clopidogrel**	NA
	**Other antiplatelet**	0
	**Dual antiplatelet**	NA
**Scantling D, 2017**	**Country**	USA
	**Single/multicenter**	Single center
	**Study design**	Retrospective
	**Mild TBI definition**	Isolated head injury with a GCS>13
	**Primary outcome**	Incidence of delayed ICH
	**Inclusion criteria**	Patients >65 years old; GCS of 14 or 15; on antithrombotic therapy; admitted to level one trauma center; received both an initial and a delayed CT of the head (12 h after admission without prompting by a change in clinical status)
	**Exclusion criteria**	Patients found to have findings of an intracranial injury on the initial CT
	**Recruitment time**	2010 – 2012
	**Patients enrolled**	234
	**Mean Age, years**	80.9
	**Male (%)**	102 (43.5)
	**Time from trauma to ED presentation**	NA
	**Time from first to second CT scan**	14 hours
	**Patients enrolled in our meta-analysis**	165
	**Reference standard for DB**	12 h RHCT
	**Aspirin**	131
	**Clopidogrel**	12
	**Other antiplatelet**	0
	**Dual antiplatelet**	22
**Stanitsas L, 2016***	**Country**	NA
	**Single/multicenter**	Single center
	**Study design**	Retrospective
	**Mild TBI definition**	NA
	**Primary outcome**	Incidence of delayed ICH

	**Inclusion criteria**	Patients with blunt mechanism of injury; taking antithrombotic agents; with evidence of cranial/facial injury; with a negative initial head CT
	**Exclusion criteria**	Patients <18 years old; patients with penetrating head trauma
	**Recruitment time**	January 2014 – December 2014
	**Patients enrolled**	71
	**Mean Age, years**	77.4
	**Male (%)**	NA
	**Time from trauma to ED presentation**	NA
	**Time from first to second CT scan**	NA
	**Patients enrolled in our meta-analysis**	40
	**Reference standard for DB**	RHCT
	**Aspirin**	30
	**Clopidogrel**	10
	**Other antiplatelet**	0
	**Dual antiplatelet**	ND
**Swap C, 2016**	**Country**	USA
	**Single/multicenter**	Multicentric
	**Study design**	Retrospective
	**Mild TBI definition**	NA
	**Primary outcome**	Incidence of delayed ICH (within 60 days) in patients receiving warfarin or clopidogrel
	**Inclusion criteria**	ED encounters for trauma receipt of a head CT scan; currently prescribed warfarin (and had an international normalized ratio above 1.2 on the day of the ED visit) or clopidogrel
	**Exclusion criteria**	NA
	**Recruitment time**	2007-2011
	**Patients enrolled**	491
	**Mean Age, years (mean±SD)**	76.6±11.9
	**Male (%)**	258 (52.5)
	**Time from trauma to ED presentation**	NA
	**Time from first to second CT scan**	4.7 days
	**Patients enrolled in our meta-analysis**	260
	**Reference standard for DB**	60 days FU from medical records
	**Aspirin**	0
	**Clopidogrel**	260
	**Other antiplatelet**	0
	**Dual antiplatelet**	0
**Tauber M, 2009**	**Country**	Austria
	**Single/multicenter**	Single center
	**Study design**	Prospective
	**Mild TBI definition**	Isolated mild head injury with a GCS score of 15
	**Primary outcome**	Incidence of delayed ICH
	**Inclusion criteria**	Patients ≥65 years old; regular low-dose therapy (100 mg/d) with aspirin (independent from the indication); isolated mild head injury with a GCS score of 15; primary negative head CT; no hypertensive irregularities (systolic blood pressure < 150 mm Hg)
	**Exclusion criteria**	Patients who received other anticoagulant medications as

		warfarin, coumarin, clopidogrel, or nonsteroidal anti-inflammatory drugs; patients with hematological or oncological diseases; moderate or severe head injuries
	**Recruitment time**	July 2007 - November 2008
	**Patients enrolled**	100
	**Mean Age, years (mean±SD)**	81±10
	**Male (%)**	39 (39)
	**Time from trauma to ED presentation**	3 hours in 91 patients<br>between 3 hours and 16 hours (average 6.4± 3.9) in 9 patients
	**Time from first to second CT scan**	19 hours ± 9 hours with a minimum 12 hours
	**Patients enrolled in our meta-analysis**	100
	**Reference standard for DB**	12-24h RHCT
	**Aspirin**	100
	**Clopidogrel**	0
	**Other antiplatelet**	0
	**Dual antiplatelet**	0
**Taylor K, 2012***	**Country**	Australia
	**Single/multicenter**	Multicentric
	**Study design**	Retrospective
	**Mild TBI definition**	NA
	**Primary outcome**	To evaluate the benefit of serial CT scans to screen for delayed ICH in patients on anticoagulant/antiplatelet therapy presenting with head injuries
	**Inclusion criteria**	Patients with head trauma; on anticoagulant/antiplatelet therapy; receiving a second CT within 48 h of a negative initial CT
	**Exclusion criteria**	NA
	**Recruitment time**	July 2010 - February 2012
	**Patients enrolled**	159
	**Mean Age, years**	NA
	**Male (%)**	NA
	**Time from trauma to ED presentation**	NA
	**Time from first to second CT scan**	Within 48 hours
	**Patients enrolled in our meta-analysis**	85
	**Reference standard for DB**	RHCT within 48 h
	**Aspirin**	52
	**Clopidogrel**	26
	**Other antiplatelet**	7
	**Dual antiplatelet**	NA

*Table A. Study characteristics. LEGEND: NA=not available; CT= computed tomography; ED= Emergency Department; GCS= Glasgow Coma Scale; LDA= low dose acetylsalicylic acid; ICH= intracranial hemorrhage; EMS = Emergency Medical Services; SD=standard deviation; IQR=interquartile range; RHCT=repeated head ct scan; FU= follow-up; *Only abstract available.*

Table B: Subgroup analysis results for the primary outcome considering different antiplatelet agents.

Study	All	ASA	Clopidogrel	DAPT
**ANTONI_2019**	1.85% (0.23-6.53%)	NA	0.00% (0.00-4.2%)	9.09% (1.12-29.16%)
**BATTLE_2017**	2.27% (0.06-12.02%)	0.00% (0.00-26.46%)	3.45% (0.09-17.76%)	NA
**CHENOWETH_2018**	0.0% (0.00-1.92%)	0.00% (0.00-2.4%)	NA	NA
**ERNSTBRUNNER_2016**	1.05% (0.29-2.66%)	1.05% (0.29-2.66%)	NA	NA
**GALLIAZZO_2019**	1.53% (0.19-5.41%)	NA	NA	NA
**GANETSKY_2017**	0.16% (0.00-0.87%)	0.18% (0.00-0.98%)	0.00% (0.00-16.11%)	0.00% (0.00-7.11%)
**HILL_2018**	3.29% (1.33-6.65%)	2.91% (0.60-8.28%)	0.00% (0.00-7.71%)	6.25% (1.73-15.24%)
**HUANG_2019**	2.52% (0.52-7.19%)	0.00% (0.00-3.66%)	15% (3.21-37.89%)	NA
**MANN_2018**	0.00% (0.00-3.18%)	0.00% (0.00-4.45%)	0.00% (0.00-21.8%)	0.00% (0.00-19.51%)
**NISHIJIMA_2012**	0.00% (0.00-1.53%)	NA	NA	NA
**PECK_2011**	0.00% (0.00-3.52%)	NA	NA	NA
**SCANTLING_2017**	1.2% (0.15-4.31%)	0.76% (0.02-4.18%)	0.00% (0.00-26.46%)	4.55% (0.12-22.84%)
**STANITSAS_2016**	0.00% (0.00-8.81%)	0.00% (0.00-11.57%)	0.00% (0.00-30.85%)	NA
**SWAP_2016**	2.31% (0.85-4.95%)	NA	NA	NA
**TAUBER_2009**	4.00% (1.1-9.93%)	4.00% (1.10-9.93%)	NA	NA
**TAYLOR_2012**	0.00% (0.00-4.25%)	0.00% (0.00-6.85%)	0.00% (0.00-13.23%)	NA
**N° studies**	**16**	**11**	**9**	**5**
**DB mean estimated risk**	**0.77% (0.23-1.52%)**	**0.22% (0.00-0.89%)**	**0.22% (0.00-2.32%)**	**2.64% (0.03-7.65%)**
**p value for heterogeneity**	**p=0.0007**	**p=0.0873**	**p=0.02224**	**p=0.1430**
**I^2 statistic**	**61%**	**39%**	**25%**	**42%**

Table B legend: results are represented as mean estimates and 95% confidence interval (in brackets). DB = delayed bleeding; all= all patients included in the analysis; ASA=acetylsalicylic acid subgroup; clopidogrel= clopidogrel subgroup, DAPT=dual antiplatelet agent subgroup; NA not assessed.
