# Supplementary material for: Incidence of delayed bleeding in patients on antiplatelet therapy after mild traumatic brain injury: a systematic review and meta-analysis
Source: Scand J Trauma Resusc Emerg Med. 2021 Aug 23;29:123. doi: 10.1186/s13049-021-00936-9 (PMC8381571; doi:10.1186/s13049-021-00936-9)
Supplement: Supplementary file 3 — Additional file 3. Forest plots about the subgroup analysis performed if considering variability in age and in the means of delayed bleeding detection (Figure A to C), and about the two sensitivity analyses performed (Figure D and E). [file 13049_2021_936_MOESM3_ESM.pdf]

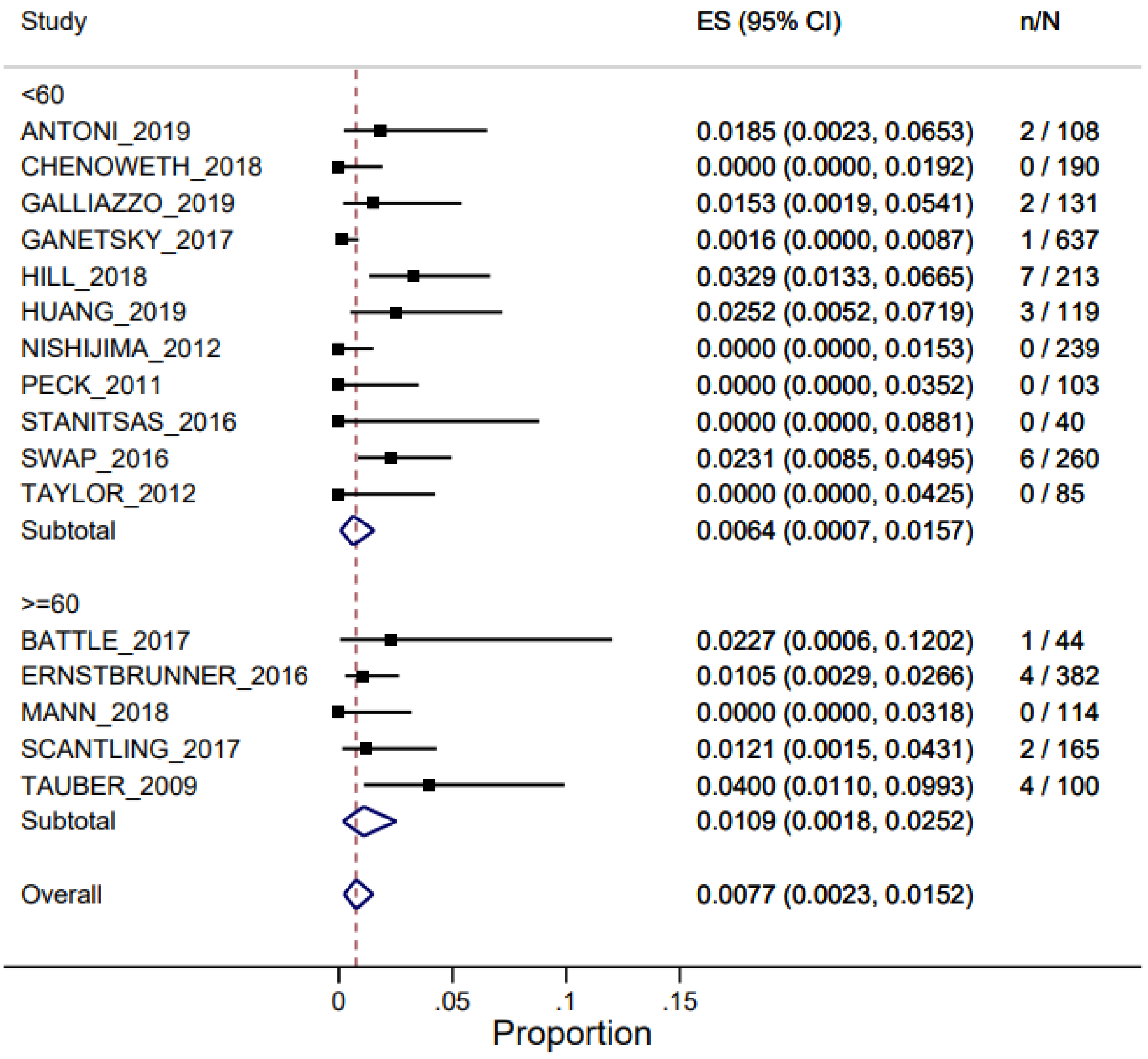

Figure A. It represents random effect meta-analysis results for the primary outcome considering age subgroup with age cut-off at 60 years. Legend, ES (95% CI): estimated mean value and 95% confidence interval; n/N: number of events/number of patients; ≥ 60 yo: included only studies enrolling patients older than 60 yo; < 60 yo: includes only studies enrolling any patients without any age restriction.

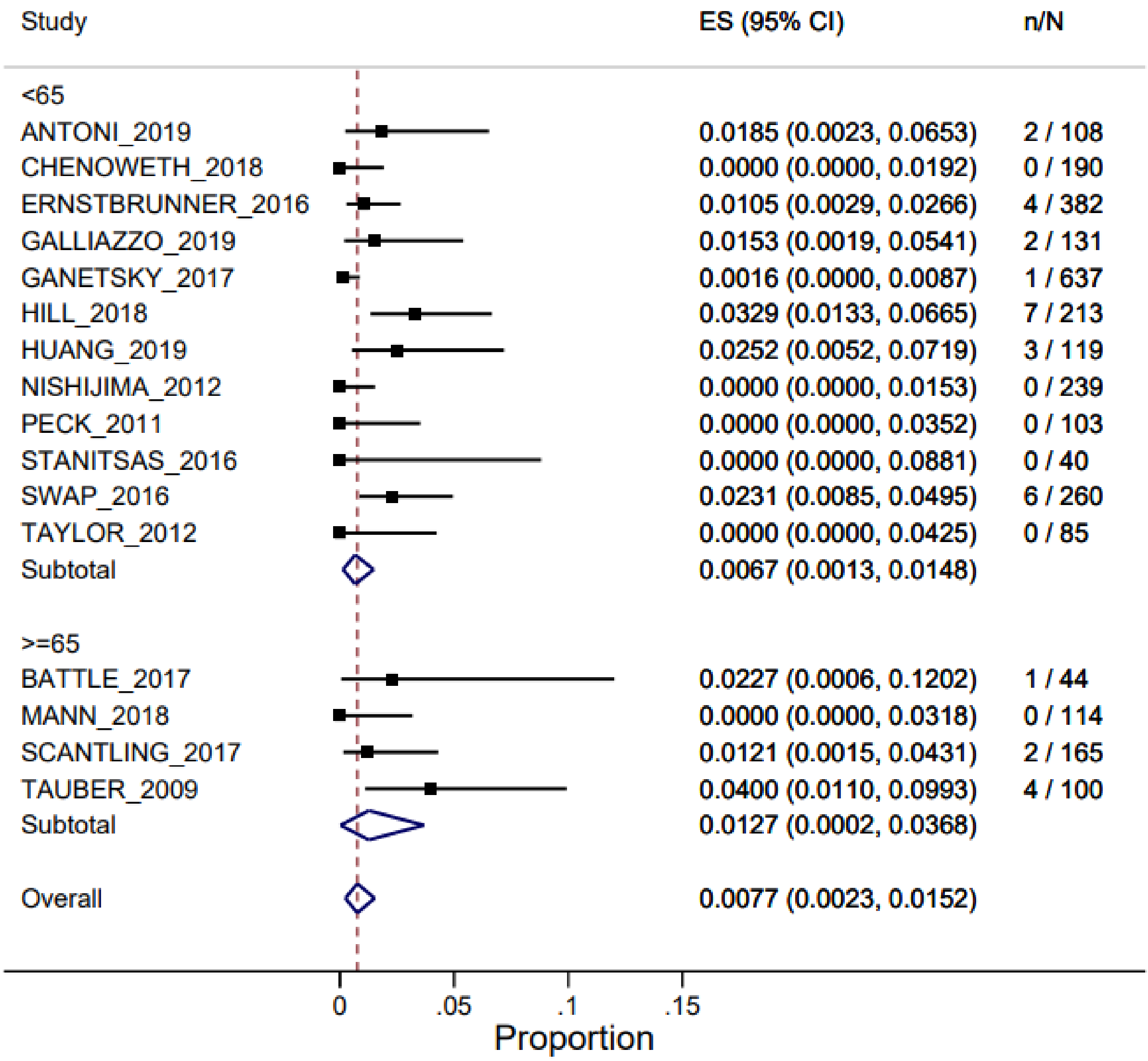

Figure B. It represents random effect meta-analysis results for the primary outcome considering age subgroup with age cut-off at 65 years. Legend, ES (95% CI): estimated mean value and 95% confidence interval; n/N: number of events/number of patients; ≥ 65 yo: included only studies enrolling patients older than 65 yo; < 65 yo: includes only studies enrolling any patients without any age restriction.

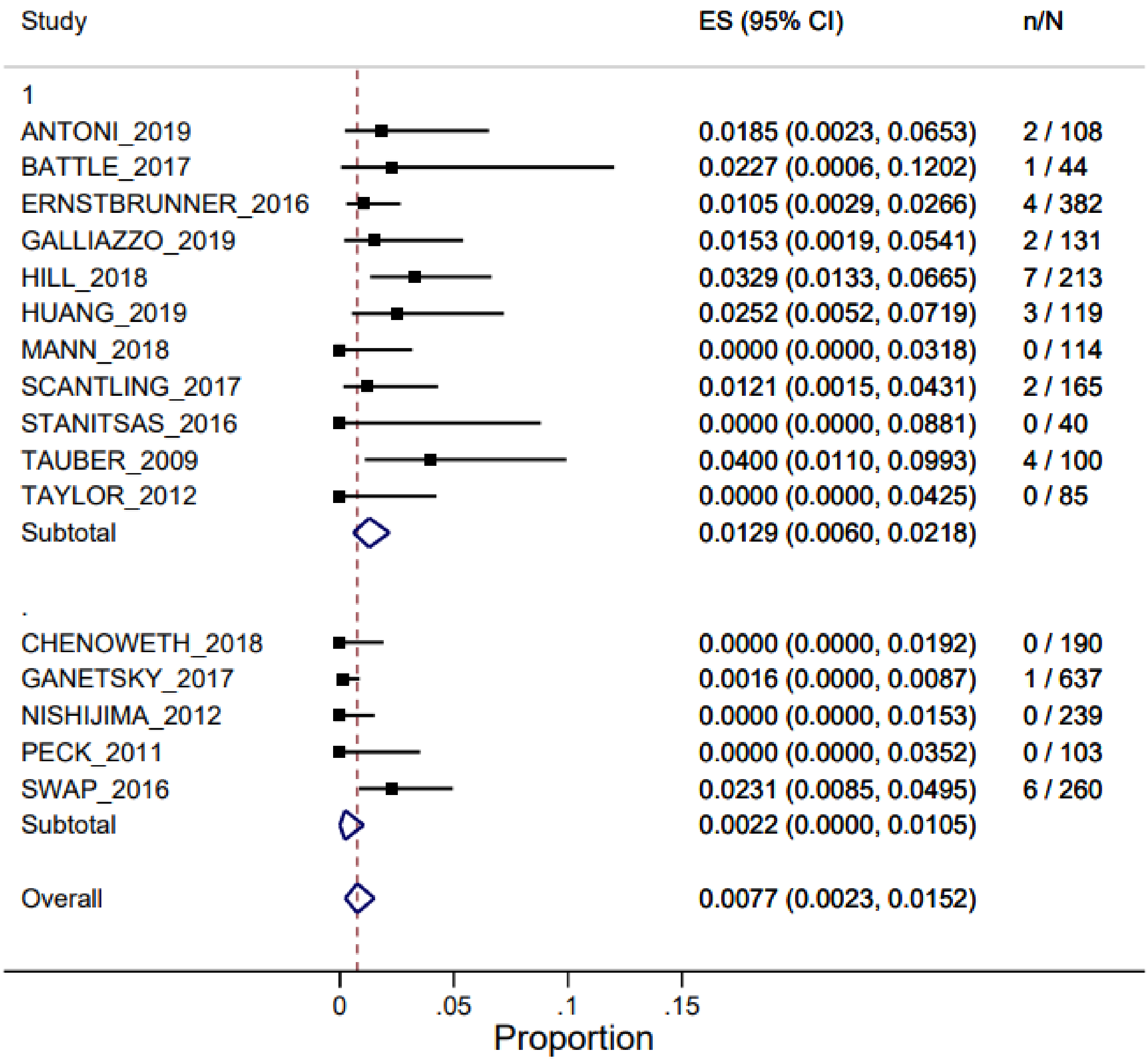

Figure C. It represents random effect meta-analysis results for the primary outcome considering subgroups by mean of outcome assessment. Legend, ES (95% CI): estimated mean value and 95% confidence interval; n/N: number of events/number of patients; 1= CT scan outcome detection subgroup; 0= follow-up outcome detection subgroup.

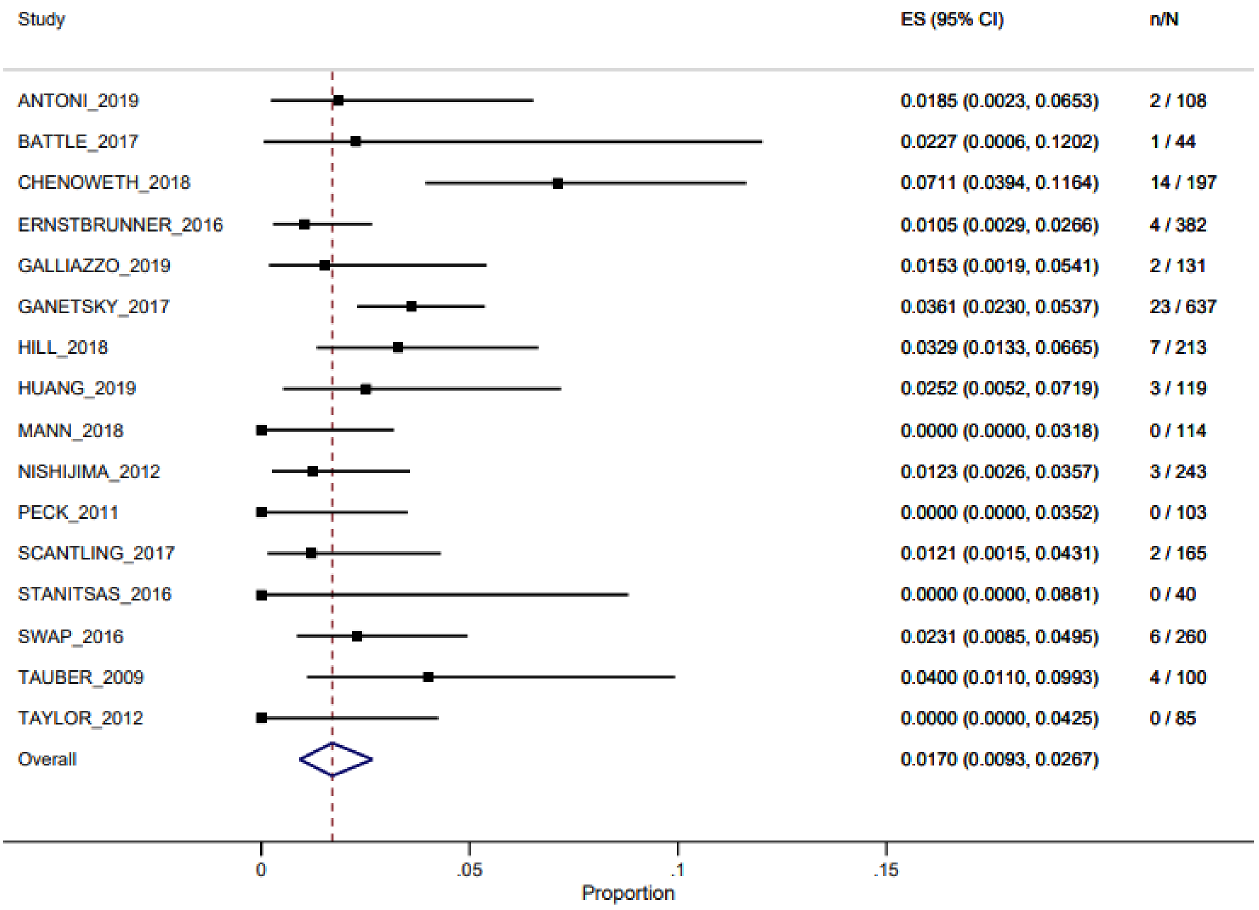

Figure D. It represents random effect meta-analysis results considering loss at follow-up or unexplained death as events. Legend, ES (95% CI): estimated mean value and 95% confidence interval; n/N: number of events/number of patients.

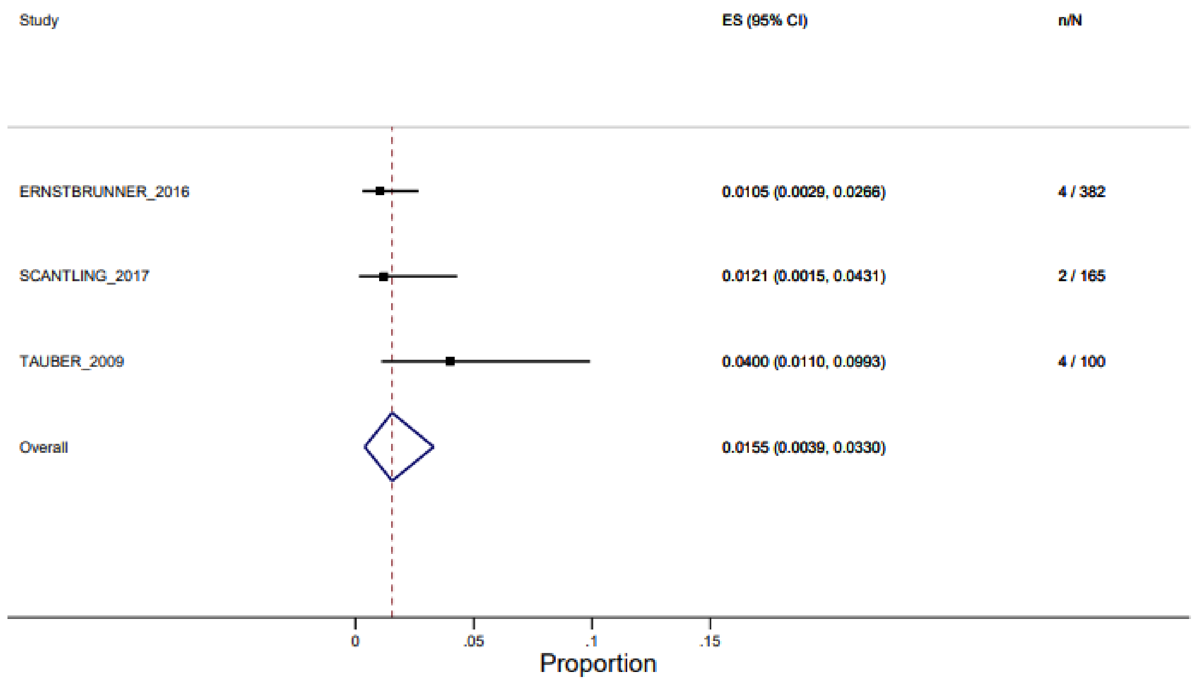

Figure E. It represents random effect meta-analysis results for the primary outcome considering mild-traumatic brain injury subgroup defined as traumatic brain injury with a presentations GCS > 13. Legend, GCS: Glasgow coma scale; ES (95% CI): estimated mean value and 95% confidence interval; n/N: number of events/number of patients.
